# EFFECTS OF GAMIFIED COGNITIVE TRAINING TO DETER THE PROGRESSION OF MILD COGNITIVE IMPAIRMENT

**DOI:** 10.1093/geroni/igad104.2098

**Published:** 2023-12-21

**Authors:** Doris S F Yu, Polly Li, Wung Wai Two, Mary Waye

**Affiliations:** The University of Hong Kong, Hong Kong, Hong Kong; The University of Hong Kong, Hong Kong, Hong Kong; Hong Kong Society for Sudoku Advancement, Hong Kong, Hong Kong; The Chinese University of Hong Kong, Hong Kong, Hong Kong

## Abstract

Mild cognitive impairment (MCI) is highly prevalent condition among older adults, and has a greater risk of progression to dementia. Even cognitive training has great efficacy to delay the onset of dementia, its time-limited offer by professional members reduces its availability. Whereas ‘gamification’ evolves as important concept to enhance treatment engagement, we developed an innovative Sudoku solving algorithm titled ‘T-code’ to assimilates the key domains of cognitive training on attention, logical and deductive reasoning. A single-blinded RCT randomized 288 older adults with MCI to receive the 24-week Sudoku Mind Activation and Revitalizing Training (SMART) Program (n=157) or the usual care (n = 131). A battery of cognitive assessments, the Memory Inventory for Chinese, the Short Form-36 were evaluated at baseline, immediately after the 12-week Sudoku training and after the 12-week home-based self-practice. Focus group interview was conducted to explore subjects’ experience and reasons for its therapeutic effects if any. The results indicated that participants in the SMART Program had greater improvements in global cognitive function, working memory, language, short- and long-term delay recalls, recognition memory, CTT 1 and subjective memory complaints immediately after the 12-week training than the control. These effects, together with executive function, were sustained till the end of the programme. The qualitative findings converged with the outcome evaluation, and improved self-confidence, more active life engagement, and enhanced mood status might sustain the cognitive benefits. This game-based cognitive training is a treatment option of high accessibility, availability and acceptance to prevent MCI from progressing to dementia.

